# Bevacizumab—Insights from EudraVigilance Database on the Assessments of the Safety Profile of Monoclonal Antibodies Used as Targeted Cancer Treatment

**DOI:** 10.3390/ph18040501

**Published:** 2025-03-30

**Authors:** Razvan Constantin Vonica, Anca Butuca, Claudiu Morgovan, Manuela Pumnea, Remus Calin Cipaian, Adina Frum, Carmen Maximiliana Dobrea, Andreea Loredana Vonica-Tincu, Aliteia-Maria Pacnejer, Steliana Ghibu, Florina Batar, Felicia Gabriela Gligor

**Affiliations:** 1 Preclinical Department, Faculty of Medicine, “Lucian Blaga” University of Sibiu, 550169 Sibiu, Romania; razvanconstantin.vonica@ulbsibiu.ro (R.C.V.); manuela-pia.pumnea@ulbsibiu.ro (M.P.); adina.frum@ulbsibiu.ro (A.F.); carmen.dobrea@ulbsibiu.ro (C.M.D.); loredana.vonica@ulbsibiu.ro (A.L.V.-T.); aliteia.pacnejer@ulbsibiu.ro (A.-M.P.); florina.batar@ulbsibiu.ro (F.B.); felicia.gligor@ulbsibiu.ro (F.G.G.); 2 Clinical Department, Faculty of Medicine, “Lucian Blaga” University of Sibiu, 550169 Sibiu, Romania; calin.cipaian@ulbsibiu.ro; 3 County Clinical Emergency Hospital of Sibiu, 2–4 Corneliu Coposu Str., 550245 Sibiu, Romania; 4 Department of Toxicology, Drug Industry, Management and Legislation, Faculty of Pharmacy, “Victor Babeş” University of Medicine and Pharmacy, 2nd Eftimie Murgu Sq., 300041 Timişoara, Romania; 5 Department of Pharmacology, Physiology and Pathophysiology, Faculty of Pharmacy, “Iuliu Haţieganu” University of Medicine and Pharmacy, 400349 Cluj-Napoca, Romania; steliana.ghibu@umfcluj.ro

**Keywords:** colon cancer, bevacizumab, monoclonal antibodies, pharmacovigilance, personalized therapy

## Abstract

**Background/Objectives:** Worldwide, colon cancer is a major cause of cancer-related mortality, with an increasing incidence influenced by genetic, environmental, and lifestyle factors. Despite advances in diagnosis and personalized treatments, challenges remain in improving patient prognosis, particularly in metastatic colorectal cancer (mCRC). Bevacizumab (BEV), a monoclonal antibody, is widely used in colorectal cancer treatment. This study aimed to analyze adverse events associated with BEV compared with other therapies based on data from the EudraVigilance (EV) database. **Methods:** A descriptive and disproportionality analysis was conducted on signals reported in the EV database related to BEV. The study included comparisons with other antineoplastic treatments, such as chemotherapy, targeted therapy, and immunotherapy. Patient demographics, severity of adverse drug reactions (ADRs), and distribution patterns were analyzed to assess the safety profile of BEV in colorectal cancer treatment. **Results:** The majority of the signals for BEV were from patients aged 18–64 years (39.42%) and 65–85 years (34.08%). Hypertension, thromboembolism, proteinuria, and gastrointestinal disorders have been the most frequently reported. Serious ADRs, including gastrointestinal perforations, hemorrhage, and arterial thromboembolism, were observed in 93.74% of Individual Case Safety Reports. BEV was associated with a higher likelihood of vascular and endocrine disorders compared with chemotherapy and other targeted therapies. Immunotherapy was linked to increased immunological ADRs, while BEV demonstrated fewer immune-related toxicities. **Conclusions:** Continuous monitoring is necessary to optimize patient management, particularly in elderly patients or those with cardiovascular comorbidities. Understanding BEV’s safety profile allows for better personalization of treatment strategies, minimizing risks while enhancing therapeutic outcomes.

## 1. Introduction

Colon cancer is one of the leading causes of cancer mortality worldwide [1], with an increasing incidence, especially in developed countries, and is influenced by a combination of genetic, environmental and lifestyle factors [2]. In 2022, more than 1.92 million [3] cases of colorectal cancer (CRC) were diagnosed, making it the third most common cancer in the world [4,5]. Early diagnosis and personalized treatments have improved patient survival, but challenges remain, especially in the case of targeted therapies [6].

The last 20 years have brought great progress in the treatment of colorectal cancer. In the case of patients with metastatic colorectal cancer (mCRC) treated with multimodality therapy, they can have a median survival of over 30 months, but the prognosis of patients with mCRC must be constantly improved [7].

Currently, there are many promising therapeutic methods, in addition to chemotherapy and molecular agents targeting the epidermal growth factor receptor (EGFR) and vascular endothelial growth factor (VEGF), which bring considerable benefits to the patient’s survival and quality of life [8]. It is also known that chemotherapy combined with targeted therapy improves the rate and efficiency of hepatectomy, thus leading to a much increased progression-free survival (PFS) and overall survival (OS) [9].

Bevacizumab (BEV) is a humanized recombinant immunoglobulin-1 (IgG1) monoclonal antibody with anti-VEGF activity [10]. It is currently used in the treatment of many types of neoplasia, especially colorectal cancer [11]. Its action is to competitively inhibit VEGF secreted by the tumor, preventing its association with receptors on neighboring endothelial cells [12]. The advantage of BEV combined with chemotherapy treatment indicated in patients with mutant RAS status in the metastatic stage may be due to an increased sensitivity that cancer cells develop but also to a more efficient distribution of cytostatics at the tumor level [13,14].

The stage at diagnosis will determine the treatment [15]. Stage IV is correlated with the presence of distant metastases, for which treatment involves combination chemotherapy regimens such as FOLFOX (leucovorin (LV), 5-fluorouracil (5-FU) and oxaliplatin (OX)) or FOLFIRI (LV, 5-FU and irinotecan (IRI)) [16] associated with anti-epidermal growth factor receptor (EGFR) agents [17,18] such as cetuximab (CET) or panitumumab (PAN) or antiangiogenic drugs (BEV or aflibercept—AFL) [19]. The goal at this stage is to prolong survival or even cure if resection of liver metastases is required [20].

Given its widespread use, over time, a series of adverse reactions (ADRs) have been identified and reported, such as hypertension, thromboembolism, proteinuria, slow wound healing, risk of fistula, infections, gastrointestinal disorders and heart failure. In terms of pulmonary toxicities associated with BEV, hemoptysis and pulmonary hemorrhage have been reported [21]. BEV is considered a safe drug, but rare events regarding lung lesions are reported in the specialized literature, for example, chronic interstitial pneumonia, alveolar hemorrhage, and acute interstitial pneumonitis [22,23].

Regarding the treatment based on cytostatics, their ADRs can vary depending on the dose, the patient’s condition and the duration of treatment. Hematological, gastrointestinal, neurological, cardiac toxicity and asthenia are the most frequently reported ADRs [24].

In addition to chemotherapy combined with monoclonal antibodies, in recent years, immunotherapy has revolutionized the treatment of CRC [25]. Immune checkpoint inhibitors (ICI) bring considerable clinical benefits to patients diagnosed with CRC, especially for patients with high microsatellite instability (MSI-H) [26].

Most CRC cases are resistant to immune checkpoint blockade (ICB) immunotherapy, with only 15% of these having microsatellite instability-high (MSI-H) [27]. The mechanisms by which this ICB resistance occurs are not fully understood. In 2017, the US Food and Drug Administration (FDA) approved nivolumab (NIV), an anti-PD-1 antibody, for the treatment of MSI-H metastatic colorectal cancer with DNA mismatch repair (dMMR) deficiency that has progressed after standard treatments with 5-FU, OX and IRI [28].

In 2020, pembrolizumab (PEM), an anti-programmed cell death 1 (PD-1) agent, was approved by the FDA as a first-line treatment for patients with metastatic CRC and MSI-H. In the clinical trial, PEM demonstrated its efficacy, significantly improving PFS to 16.5 months from 8.2 months. In terms of safety profile, the drug was better tolerated and had fewer ADRs than chemotherapy as a first-line treatment in the clinical trial [29].

As for ADRs regarding ICI treatment, they can affect any organ through the mechanism of stimulation of the immune system, such as skin (rash, itching, vitiligo, Stevens-Johnson syndrome, etc.), autoimmune colitis, autoimmune hepatitis, hypophysitis, hypothyroidism or hyperthyroidism, diabetes mellitus, adrenal insufficiency, pneumonitis, autoimmune nephritis, myocarditis, pericarditis, encephalitis, peripheral neuropathies, etc. [30,31].

Currently, various personalized therapeutic strategies are being analyzed for patients diagnosed with CRC, such as chemotherapy, targeted therapies based on specific biomarkers, immunotherapy [32]. Significant challenges in choosing individualized treatment are currently identified [33].

This study discusses the reality of personalized treatments based on data uploaded to the EudraVigilance (EV) database, regarding the risk of ADRs of BEV in particular but also their comparison with other therapies.

BEV treatment in CRC brings out significant benefits but also risks, and the analysis of ADRs reported in the EV database provides a valuable insight into its safety compared to other therapies, such as chemotherapy, other monoclonal antibodies and immunotherapy. The study of these ADRs helps to understand the safety profile of BEV, thus allowing an individual selection of treatment according to the characteristics of the patients. Compared with other therapeutic options, this type of analysis can support more personalized and safer treatment strategies, optimizing patient management.

## 2. Results

### 2.1. Descriptive Analysis

The total number of ICSRs reported in the EV database until 1 December 2024 was 59.693, most of them for the 18–64 years (n = 23,529, 39.42%) and 65–84 years categories (n = 20,345, 34.08%) (Table 1). The distribution of ICSRs by sex, origin or reporter category varied. Thus, 49.80% of cases have been reported in the female group (n = 29,729), and 41.75% have been reported in the male group (n = 24,921). More than two-thirds of cases were reported outside the European Economic Area (EEA) (n = 41,101). Not least 93.93% of ICSRs (n = 56,072) have been filled by healthcare professionals (HP).

Table 2 presents the distribution of reports by SOC. According to these data, the SOCs with the higher frequency of occurrence are “Gastrointestinal disorders” (n = 13,456, 12.57%), “General disorders and administration site conditions” (n = 13,360, 12.48%), “Blood and lymphatic system disorders” (n = 8184, 7.64%), “Nervous system disorders” (n = 7851, 7.33%) and “Vascular disorders” (n = 7285, 6.80%).

For 56.6% of ICSRs submitted in EV between 2005 and 2024, no other concomitant drugs have been reported. But, in 43.4% of cases, other drugs were used associated with BEV.

### 2.2. Disproportionality Analysis

#### 2.2.1. Systemic Therapy

**Figure 1:**
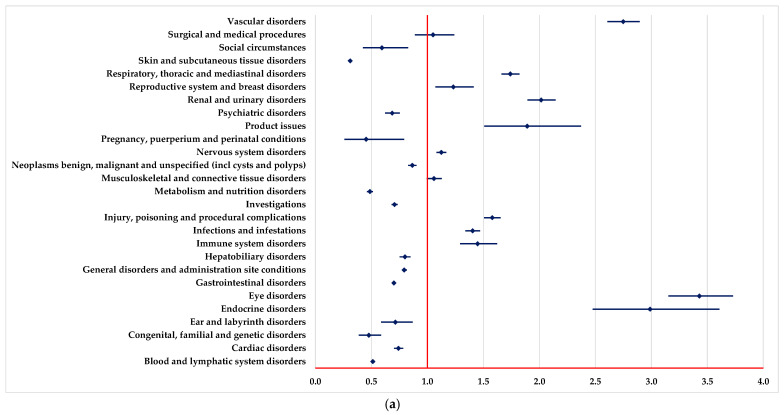

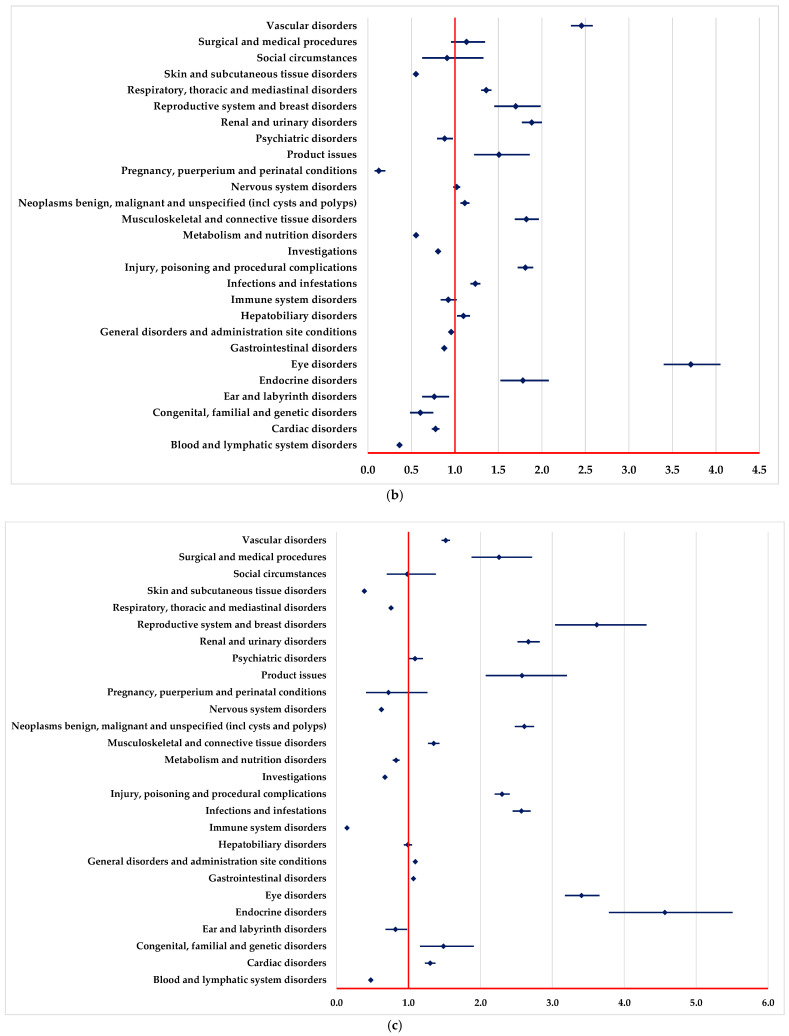

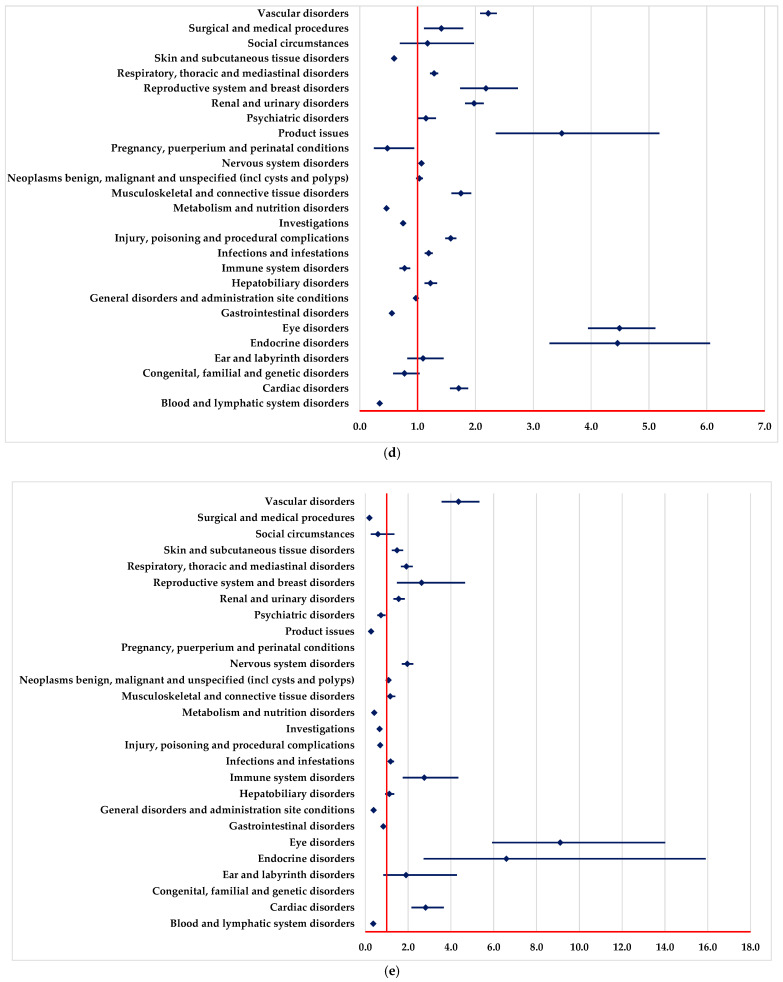
Disproportionality analysis compared to drugs used as systemic therapy. (**a**)—capecitabine; (**b**)—5-fluorouracil; (**c**)—oxaliplatin; (**d**)—irinotecan; (**e**)—trifluridine-tipiracil (TFT).

PTs found in some SOCs have been reported for BEV with a higher probability than drugs used as systemic therapy (Figure 1), including vascular disorders, eye disorders, endocrine disorders, infections and infestations, and renal and urinary disorders.

On the other hand, by comparison with systemic therapy, a lower probability of reporting ADRs from some SOCs could be observed for BEV: blood and lymphatic system disorders and metabolism and nutrition disorders (Figure 1).

#### 2.2.2. Targeted Therapy

**Figure 2:**
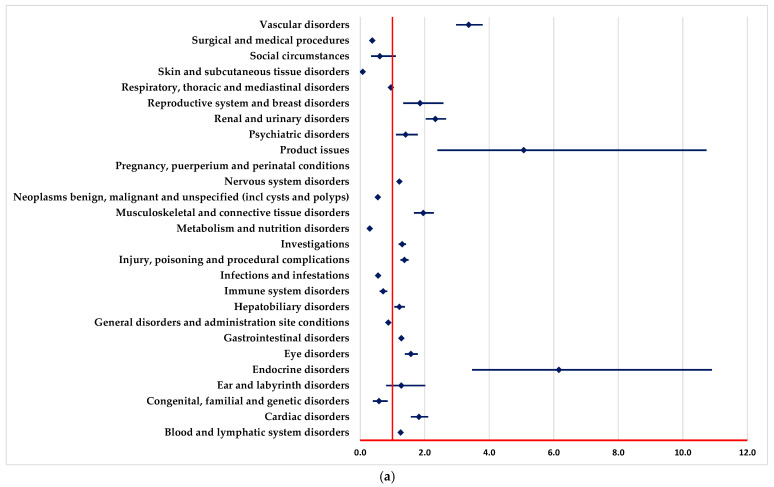

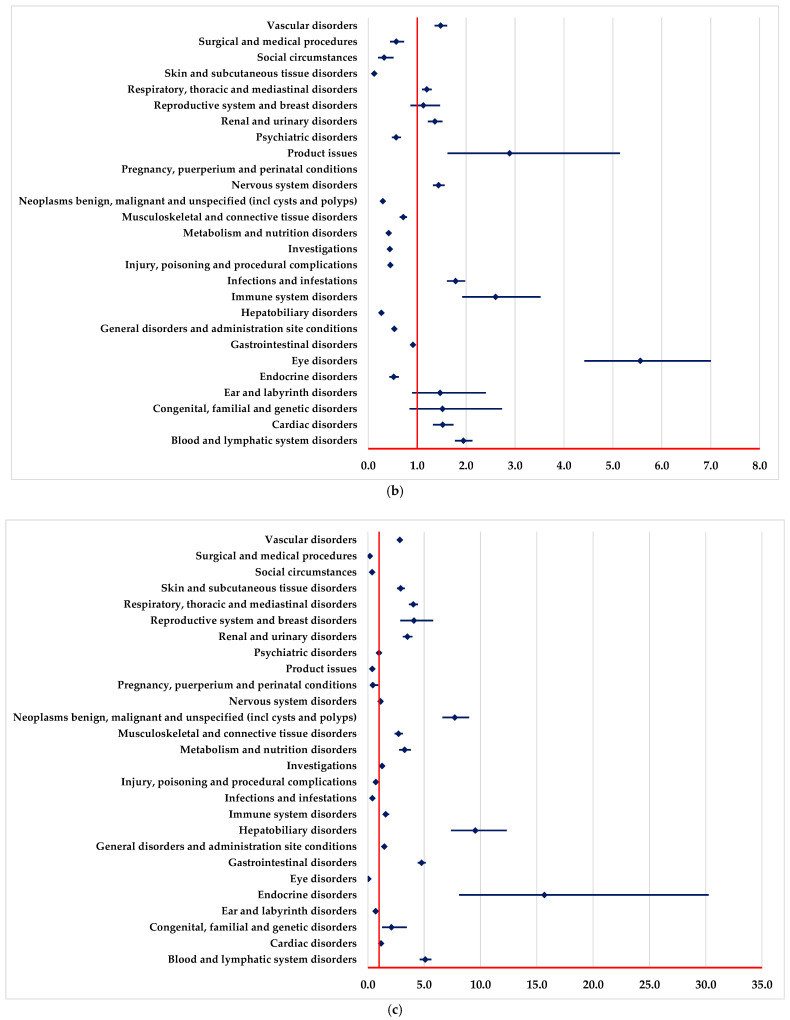

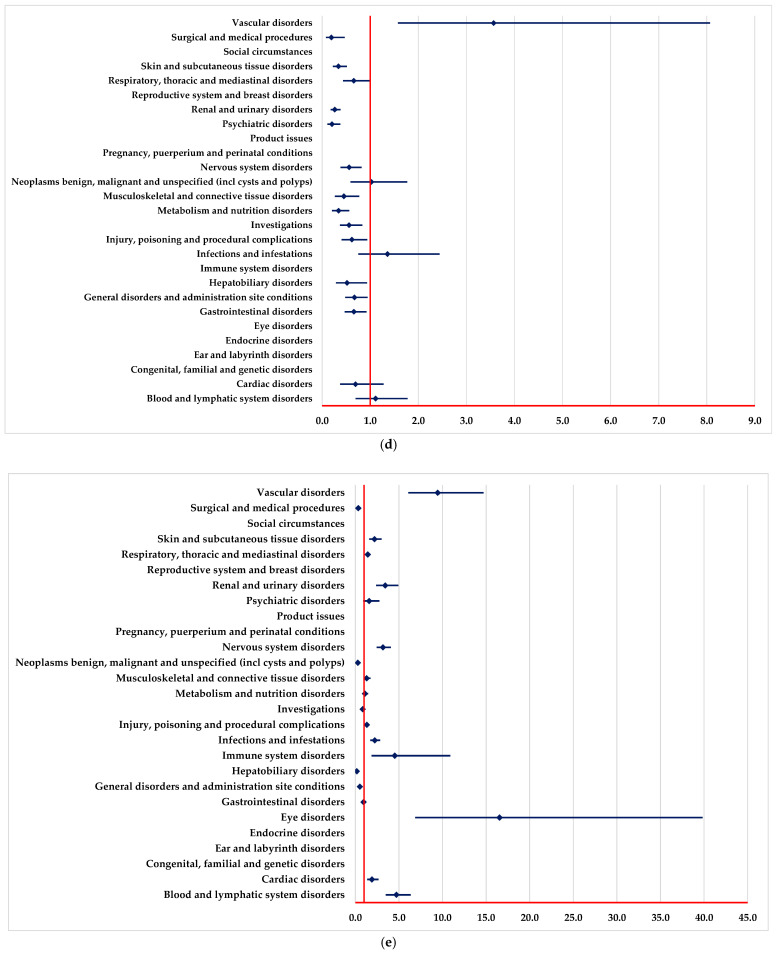
Disproportionality analysis compared to drugs used as targeted therapy. (**a**)—panitumumab; (**b**)—regorafenib; (**c**)—aflibercept; (**d**)—adagrasib; (**e**)—sotorasib.

A higher probability of reporting ADRs in “Blood and lymphatic system disorders” and “Vascular disorders” SOCs was registered in comparison to PAN, REG, AFL and SOT (Figure 2a–c,e).

ADRs included in “Infections and infestations” SOC are reported to have a higher probability for BEV in comparison to REG and SOT (Figure 2b,e).

A lower probability of reporting in the “Surgical and medical procedures” SOC was observed compared with PAN, REG, AFL, ADA and SOT (Figure 2a,c–e). On the other hand, ADRs from numerous SOCs are reported more frequently for BEV than for PAN (Figure 2a), such as cardiac disorders, eye disorders, nervous system disorders, and renal, urinary, and vascular disorders.

In the following SOCs, ADRs are reported with a lower probability for BEV than PAN (Figure 2a), among these we list metabolism and nutrition disorders, immune system disorders, infections and infestations, and skin and subcutaneous tissue disorders.

#### 2.2.3. Immunotherapy (PEM, NIV, DOS)

**Figure 3:**
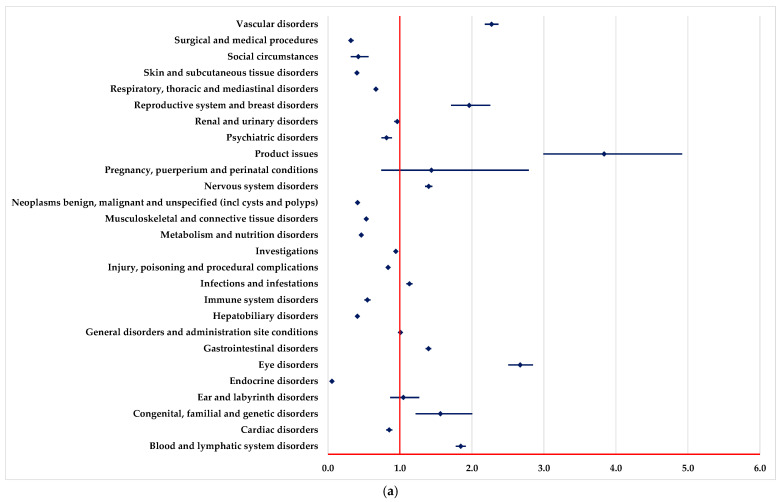

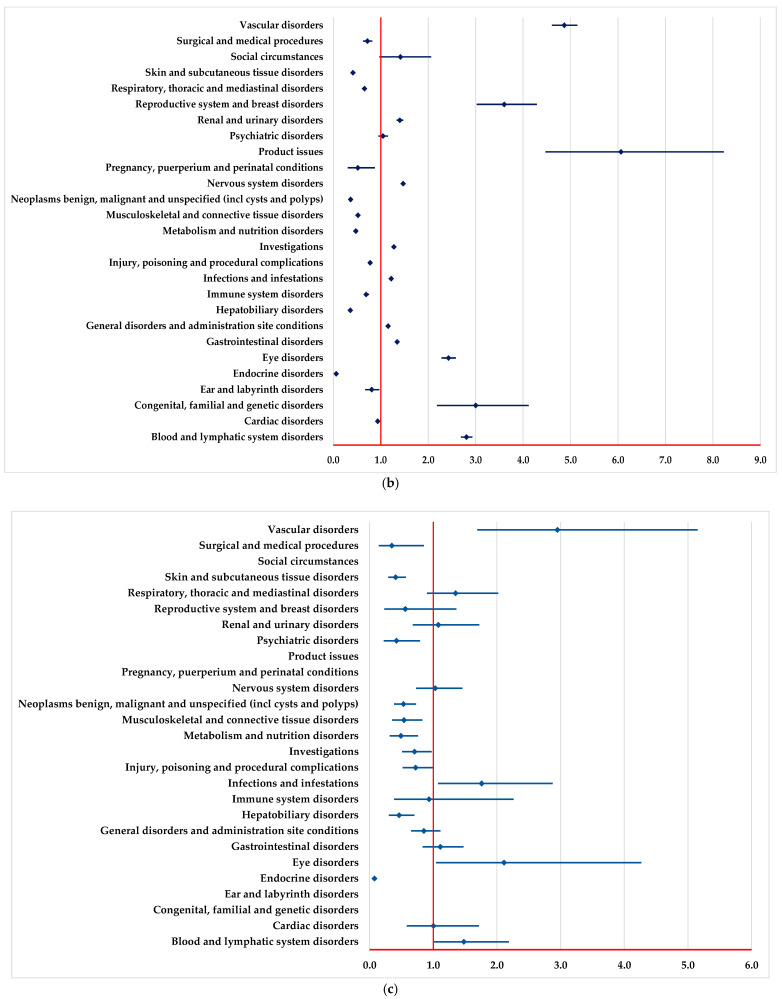
Disproportionality analysis compared with drugs used as immunotherapy. (**a**)—pembrolizumab; (**b**)—nivolumab; (**c**)—dostarlimab.

By comparison with PEM, NIV and DOS, a higher probability of reporting ADRs from the following SOCs could be observed (Figure 3): blood and lymphatic system disorders, vascular disorders, infections and infestations, and eye disorders. Moreover, by comparison with PEM and NIV, a higher probability of reporting ADRs from some SOCs is observed (Figure 3b), including nervous system disorders, reproductive system and breast disorders.

A lower probability of reporting ADRs from some SOCs is observed by comparison with PEM, NIV and DOS (Figure 3), among which we mention endocrine disorders, hepatobiliary disorders, skin and subcutaneous tissue disorders, surgical and medical procedures. Also, by comparison with PEM and NIV, a lower probability of reporting ADRs from some SOCs is observed (Figure 3a,b): immune system disorders and respiratory, thoracic and mediastinal disorders.

## 3. Discussion

In our study, we analyzed the ADRs reported in the EV database for BEV while using both descriptive and disproportionality analysis. The severity and characteristics of ADRs associated with BEV were evaluated in comparison with antineoplastic treatments used in CRC from different therapeutic classes, such as chemotherapy, targeted therapy and immunotherapy. The descriptive analysis regarding BEV provides information on the typology of ADRs and the characteristics of the patients. It is observed that the majority of reports in the EV database were registered in the 18–64 age group, representing 39.42%, and the 65–85 age group, a percentage of 34.08%. Thus, it is highlighted that BEV is used more frequently in neoplasms of the adult population but also in age groups vulnerable to ADRs.

Different studies suggest that the choice between monotherapy and combination therapy should be made adaptively according to the individual patient profile. In patients with cardiovascular comorbidities or who are elderly, it is recommended that treatment be less aggressive, thus avoiding the severe complications associated with BEV [11]. The CALGB 80405 trial compared the efficacy of FOLFIRI or FOLFOX with BEV versus cetuximab in metastatic CRC. The results indicated that BEV improved progression-free survival but was associated with a higher risk of hypertension and thromboembolic events [8]. Molecular markers, such as VEGF expression, can guide the choice of optimal and personalized treatment with good therapeutic outcomes and low risk of adverse effects [34]. Hapani et al. demonstrated in a recent meta-analysis that patients receiving BEV in combination with chemotherapy had a higher incidence of severe hypertension (up to 40%) compared with patients treated with chemotherapy alone (approximately 15%) [35]. The FIRE-3 study indicated that although the addition of BEV to chemotherapy did not significantly increase treatment-related mortality, vascular and hemorrhagic toxicities were more frequent in this group [36]. Comparative analyses highlight that combination therapy with BEV and immunotherapy may provide significant therapeutic benefits for patients with MSI-H tumors based on the synergistic effect in blocking angiogenesis and stimulating the antitumor immune response [37]. However, patient information and special attention to the increased risk of immune ADRs, including pneumonitis and autoimmune colitis, are necessary [38].

The mechanism of action of BEV has guided its use in tumors that associate a complex process of angiogenesis [39]. Specifically, the involvement of the VEGF signalling pathway in cancer progression has highlighted the correlation of increased levels of intratumoral VEGF expression with an unfavorable prognosis or a more aggressive evolution of the disease in various oncological pathologies, such as mCRC, non-small cell lung cancer (NSCLC), metastatic breast cancer (mBC), glioblastoma multiforme (GBM) and ovarian cancer (OC). Clinical studies conducted for BEV target a wide range of indications and have demonstrated its clinical benefits in mCRC and NSCLC, as well as mBC, GBM, OC and cervical cancer (CC) [40,41,42,43]. As for the gender of the patients included in the reports, it is observed that a proportion of 49.8% were women, which demonstrates the concordance with the increased prevalence of some types of cancer among women, such as breast, ovarian and uterine cancer, where treatment with BEV is recommended and plays an important role in adjuvant or second-line treatment [44]. The results suggested that ADRs are reported more frequently in the non-EEA than in the European region, reflecting a possible difference in regulation and sensitivity to drug safety in the two areas analyzed. Gender-based safety differences were analyzed in a large epidemiological study conducted by Liu et al., which showed that women are at higher risk of severe gastrointestinal toxicity under BEV treatment compared to men. This result indicates the need for personalized therapeutic approaches tailored to the individual patient profile [45].

In the specialized literature, the most frequent ADRs identified in the treatment of BEV include arterial hypertension, fatigue, asthenia, diarrhea and abdominal pain [46]. Also, the treatment with BEV is associated with the development of proteinuria, with a higher incidence being recorded in renal cancer, the severity of which can vary up to nephrotic syndrome [47]. As a consequence, it is essential to monitor proteinuria in patients undergoing treatment with BEV [48].

The highest frequency of ADRs’ occurrence in SOCs were related to “Gastrointestinal disorders” (12.57%), “General disorders and administration site conditions” (12.48%) and “Blood and lymphatic system disorders” (7.64%). This could be correlated with the mechanism of action of BEV, which is a humanized monoclonal antibody that binds to all soluble and circulating forms of VEGF-A [33]. Through this interaction, BEV blocks the binding of VEGF-A to VEGFR receptors, which prevents the activation of VEGF-associated signaling pathways and stimulates the formation of new blood vessels [49]. In vivo experiments have shown that this drug inhibits vascular development, causes regression of newly formed blood vessels and contributes to the normalization of vascular networks, thus facilitating the administration of cytotoxic chemotherapy [50]. The most common serious adverse effects identified in a study by Josep Garcia et al. included gastrointestinal perforations, hemorrhage and arterial thromboembolism [51]. The highest risk for gastrointestinal perforations was observed to be associated with CRC, inflammatory bowel disease, anti-inflammatory medications and abdominal surgery [52]. Drug interactions are another essential factor to consider. The study conducted by Tianqi Gu et al. (2023) demonstrated that concomitant administration of BEV with immune checkpoint inhibitors (e.g., PEM) significantly increases the risk of hepatotoxicity and autoimmune colitis. These findings highlight the importance of close monitoring in combination therapy [53]. Serious hemorrhagic events are identified primarily in NSCLC or in patients with brain metastases [54]. Arterial thromboembolism has an increased incidence in all locations treated with BEV and includes cerebrovascular accidents, acute myocardial infarction, transient ischemic attacks and other arterial thromboembolic reactions [55]. Thromboembolic events were also associated with diabetes mellitus and age over 65 years old [56]. Regarding neurological toxicity, Kim et al. identified an association between BEV administration and an increased incidence of posterior reversible encephalopathy syndrome (PRES). This phenomenon was observed particularly in patients with severe hypertension or a history of cerebrovascular disease, suggesting the need for rigorous screening before initiating treatment [57]. BEV is generally well tolerated in a wide range of tumors [58]. Clinical experience and post-marketing monitoring have improved the knowledge regarding its safety profile. Moreover, adverse events of BEV could be well manageable. A distribution of serious ADRs was observed in a proportion of 93.74% of ICSRs reported in EV, suggesting that treatment with BEV requires continuous monitoring to prevent and treat these ADRs.

In addition, patients with diabetes may be at increased risk of complications during BEV treatment. Sparks et al. reported that diabetic patients have a higher incidence of thromboembolic events and peripheral vascular complications, suggesting the need for specific management strategies for this patient population [59].

Compared to systemic chemotherapy, BEV was associated with a higher likelihood of reporting ADRs for “Vascular disorders”, “Endocrine disorders” and “Eye disorders”. This is consistent with the mechanism of action of BEV, which inhibits angiogenesis and, therefore, may cause vascular and ocular adverse effects such as hypertension, venous thrombosis and retinal hemorrhages. On the other hand, a low reporting ratio was also observed regarding the hematological toxicity of BEV compared to systemic chemotherapy, which has a different mechanism of action. Thus, conventional chemotherapy frequently causes neutropenia, thrombocytopenia and anemia [60].

Regarding targeted therapy, BEV has more ADR reports for “Blood and lymphatic system disorders”, “Vascular disorders” and “Infections and infestations”. In particular, BEV has been reported with a significantly higher probability of inducing vascular disorders and infections, which is in line with its well-known side effects, such as thrombosis and partial immunosuppression. Compared with REG, BEV stands out in reports of ocular and cardiovascular disorders, thus highlighting the specificity of ADRs depending on the particularities of the mechanism of action of each agent. In a study that compared immunotherapy strategies, frequent pneumonitis, colitis and thyroid endocrine disorders were identified, and for BEV, ADRs related to the vascular and hematological systems, including thrombosis and bleeding, were more frequently reported [61]. In addition to the important role in angiogenesis, BEV has been shown to have an angiogenesis-independent role in immune modulation, contributing to the suppression of adaptive immunity at several stages of the cancer immune cycle [62].

Compared with previous guidelines, the new recommendations emphasize the use of inflammatory biomarkers for early detection of immune toxicities and more specific intervention strategies. For example, in autoimmune colitis, early initiation of moderate-dose corticosteroid therapy is recommended and switching to immunosuppressive agents, such as infliximab or vedolizumab, is recommended in refractory cases [61]. In addition, the ESMO 2022 guidelines emphasize the importance of closer monitoring of thyroid and pituitary function during ICI treatment, given the increased incidence of immunologically induced hypothyroidism and hypophysitis [38].

A number of clinical trials, such as the CALGB/SWOG 80405 and FIRE-3 trials, have demonstrated the safety of BEV therapy. These studies showed that patients with metastatic CRC who received chemotherapy in combination with BEV had an improved median survival compared with those treated with chemotherapy alone [63,64]. The risk of thromboembolic events was significantly higher in the BEV group, suggesting the need for close monitoring of these patients [39]. A meta-analysis of randomized controlled trials indicated that the addition of BEV to standard chemotherapy regimens increases the incidence of severe hypertension and vascular complications compared with chemotherapy alone [65]. Analysis of the TML trial (ML18147) showed that the continued use of BEV in subsequent lines of treatment was associated with a survival benefit but also with a higher incidence of severe adverse events, including bleeding and gastrointestinal perforation [37]. Given the relationship between angiogenesis and immunosuppression, the association between immune checkpoint inhibitors and BEV could have a synergic effect. Thus, further study could show more clinical benefits [66]. BEV was also less likely to induce immunological ADRs, which may be attributed to the fundamental difference in how these treatments intervene in the immune system. The results of this study emphasize the need for careful monitoring of patients treated with BEV, given the prevalence of serious ADRs, especially those of the vascular and hematological systems. For example, patients should be carefully monitored for signs of hypertension and thrombosis, especially those with cardiovascular risk factors [67]. Compared with BEV, EGFR inhibitors (e.g., cetuximab) appear to be better tolerated in older patients, with a lower incidence of thrombotic events but a higher risk of severe skin reactions [37].

Several studies have shown that elderly patients are at higher risk of thromboembolic events under BEV treatment compared with younger patients. A meta-analysis by Ferrara et al. (2022) showed that patients over 70 years of age have a two-fold higher incidence of arterial thrombosis compared with younger patients, which requires closer monitoring and possible dose adjustment [68]. Zhouling et al. highlighted that elderly patients treated with BEV have an increased risk of severe hypertension, which can lead to significant cardiovascular complications. Regular monitoring of blood pressure and early initiation of antihypertensive treatment are recommended for this category of patients [69].

Another landmark study by Price et al. (2012) indicated that gastrointestinal bleeding and intestinal perforation are more common in patients over 65 years of age receiving BEV. This may be explained by the fragility of blood vessels in older patients and the presence of pre-existing gastrointestinal comorbidities [70].

Data from the FIRE-3 trial suggest that older patients receiving combination therapy (chemotherapy + BEV) have comparable therapeutic benefit to younger patients but have a higher incidence of severe adverse effects, including thromboembolic events and hypertension [63].

Regular ocular monitoring is also essential for the early identification of complications such as retinal hemorrhages, which can lead to vision loss [71]. It is important for physicians to adapt treatment strategies and optimize therapeutic interventions to minimize the risks associated with BEV, especially in elderly patients or those with cardiovascular comorbidities [72]. An important aspect of the use of BEV is the monitoring and prevention of severe ADRs, which can significantly influence the patient’s prognosis:

(i) Patients with a history of inflammatory bowel disease or recent abdominal surgery require careful surveillance for intestinal perforation. The use of inflammatory biomarkers, such as CRP (C-reactive protein) and fecal calprotectin, can detect gastrointestinal complications [64]. Prophylactic administration of proton pump inhibitors (PPIs) is also recommended to protect the gastrointestinal mucosa. In case of a confirmed perforation, BEV treatment should be discontinued immediately, and the patient should receive intensive support and undergo emergency surgery [73];
(ii) It is important to regularly monitor hematological and coagulation parameters (INR, PT, aPTT) in order to prevent and manage severe bleeding [39]. It is recommended to avoid concomitant oral anticoagulants except in strictly indicated cases and under close monitoring. In the event of a severe bleeding episode, treatment should be stopped immediately, and the patient should be stabilized with hemostatic, blood transfusion and, if necessary, surgery [37];
(iii) Regarding BEV-induced hypertension, patients should be assessed before beginning the treatment, and those with pre-existing hypertension should receive an adjusted antihypertensive treatment regimen [38]. Blood pressure monitoring should be performed weekly during the first two months of treatment and monthly thereafter. First-line treatment includes angiotensin-converting enzyme (ACE) inhibitors and calcium channel blockers [74]. In severe cases of hypertension (>180/110 mmHg), BEV treatment should be temporarily suspended until blood pressure is stabilized [75];
(iv) In the management of thromboembolic events, pre-treatment screening for thrombotic risk factors, such as a history of deep vein thrombosis or antiphospholipid syndrome, is recommended. For patients with high-risk, prophylactic anticoagulant administration should be performed according to international guidelines [75]. In the event of a major arterial thromboembolic event, such as stroke or myocardial infarction, BEV should be permanently discontinued, and the patient should receive long-term anticoagulant therapy [68].

### Limitations of the Study

Although our study, which was based on the data mining technique from the EV database, brought several important aspects in the identification and evaluation of the safety profile of BEV, there were also some limitations. First, the EV database is a random (i.e., selective, incomplete, inaccurate and unverified) and voluntary reporting system of ADRs that may include some biases that cannot be eliminated. Thus, a limitation would be due to the design of the study that only allowed analysis of the events presented as PT in the ICSR. Moreover, this study relies solely on the EV database, which may introduce reporting biases. Being a spontaneous self-reporting system, controlling the quality of the reports is affected and cannot be guaranteed. Even if it has limited control over the accuracy of the information, EMA improves the quality of this information through continuous review. The accuracy of the data included in the ICSR depends primarily on the reporter. Thus, certain critical information regarding clinical characteristics, comorbidities, concomitantly administered drugs and treatment results may be inconsistent or even absent from these reports [76]. In this situation, it is difficult to take into account certain factors such as dose, duration of use, comorbidities, drug combinations and other factors that may influence the occurrence of ADRs.

Secondly, the EV database contains only cases with adverse events, and the incidence rate cannot be calculated due to the lack of general data on drug consumption, i.e., the lack of a denominator of drug exposure, as well as underreporting. The phenomenon of underreporting is a well-known limitation in such analyses, as it can introduce biased reporting, thus limiting the ability to estimate the actual incidence of adverse events. Failure to report an adverse event that occurred during treatment can have multiple causes, such as ignorance, guilt, mistrust, inadequate risk perception and lack of awareness of the importance of pharmacovigilance. Regarding the variability of spontaneous data reported in EV, EMA noted a difference between different regions or countries due to the differences between the existence and effectiveness of various promotion campaigns [77]. Finally, the disproportionality analysis based on EV did not quantify risk or causality but only showed an assessment of the strength of the signals.

Despite these limitations, our study provides a comprehensive post-marketing analysis of drug safety using real-world data from a large and diverse population based on spontaneous reporting systems. Furthermore, despite its inherent limitations, the disproportionality analysis is a validated and used method in post-marketing surveillance of drug safety. Moreover, further prospective studies should be performed to assess a causal relationship between the use of BEV and the reported ADRs and to provide a complete and accurate safety profile of the analyzed drugs.

## 4. Material and Methods

### 4.1. Study Design

A retrospective pharmacovigilance study regarding the ADRs reported in EV database for BEV until 1 Decembre 2024 was conducted. Data used for descriptive and disproportionality analysis were extracted on 4 December 2024 from EV portal [78].

### 4.2. Material

The Individual Case Safety Report (ICSR) is used to report ADRs in EV. Four general characteristics are collected in ICSR: age, sex, origin or reporter category. The age could be reported in the following groups: 0–1 month, 2 months–2 years, 3–11 years, 12–17 years, 18–64 years, 65–85 years, more than 85 years or not specified (NS). There are three categories for reporting the sex (male, female or NS), origin (European Economic Area—EEA, non-EEA or NS) and reporter category (healthcare professional—HP, non-HP or NS). The Medical Dictionary for Regulatory Activities (MedDRA) organized the preferred terms used for reporting ADRs in 27 system organ classes (SOCs) [78,79].

### 4.3. Descriptive and Disproportionality Analysis

Descriptive analysis included an evaluation of general characteristics (age category, sex, origin of report, reporter) presented in ICSRs. The next step was analyzing the distribution of reports with ADRs associated with BEV by SOCs.

Using the disproportionality analysis enables the identification of the similarities in reporting of ADRs. In order to perform this analysis, the European Medicine Agency (EMA) recommends a specific indicator (Reporting Odds Ratio—ROR). ROR could be calculated through the comparison with drugs from the same therapeutic areas, and the signal is disproportionate if a minimum of five cases are reported and the lower limit of 95% confidence interval (95% CI) is higher than 1 [78,79].

In the present study, the disproportionality analysis of the ADRs grouped in the 27 SOCs was performed for BEV through the comparison with different drugs used in colorectal cancer:

(i) therapy: capecitabine (CAP); 5-fluorouracil (5-FU); oxaliplatin (OXA); irinotecan (IRI); trifluridine-tipiracil (TFT);
(ii) targeted therapy: adagrasib (ADA); aflibercept (AFL); panitumumab (PAN); regorafenib (REG); sotorasib (SOT);
(iii) immunotherapy: dostarlimab (DOS); nivolumab (NIV); pembrolizumab (PEM).

MedCalc application has been used to calculate the ROR and 95% CI [80].

### 4.4. Ethics

The present study does not contain any personal information. Thus, no ethics approval was necessary [81].

## 5. Conclusions

The results of this study highlight the importance of pharmacovigilance in identifying and evaluating the complex safety profile of BEV. Using the data uploaded to the EV database, we can report the increased frequency of serious ADRs, especially at the vascular and hematological levels, which are characteristic of the mechanism of action of this drug. Compared with the studied antineoplastic treatments, BEV presents a distinct pattern of ADRs, namely the high probabilities of reporting vascular, infectious and ocular disorders, but with a low risk of hematological toxicity compared with chemotherapy. The results of the study highlight the relevance of developing BEV safety profiles and the importance of a therapeutic management strategy for the patient.

The present study shows the need for vigilant monitoring of patients receiving BEV treatment, especially patients in the advanced age group with associated comorbidities, for the prompt detection and management of potentially severe ADRs. In clinical practice, the continuous surveillance of cardiovascular, ocular and hematological parameters becomes essential, along with adjustment of therapeutic regimens and multidisciplinary collaboration to optimize treatment. Based on solid evidence of the efficacy of BEV in combination with chemotherapy, as well as emerging management indicating therapeutic outcomes when combined with innovative therapies and cancer immunotherapy, it is anticipated that BEV will continue to play a pivotal role in cancer pathology.

## Figures and Tables

**Table 1:** General characteristics of ICSRs reported in the EudraVigilance database for bevacizumab. EEA—European Economic Area; HP—healthcare professional; ICSR—Individual Case Safety Report; NS—not specified.

	n	%
Total ICSRs	59,693	100.00%
Age category		
NS	14,165	23.73%
0–1 Month	13	0.02%
2 Months–2 Years	72	0.12%
3–11 Years	285	0.48%
12–17 Years	193	0.32%
18–64 Years	23,529	39.42%
65–85 Years	20,345	34.08%
More than 85 Years	1091	1.83%
Sex		
Female	29,729	49.80%
Male	24,921	41.75%
NS	5043	8.45%
Origin		
EEA	18,592	31.15%
Non-EEA	41,101	68.85%
NS	0	0.00%
Reporter category		
HP	56,072	93.93%
Non-HP	3600	6.03%
NS	21	0.04%

**Table 2:** Distribution of reports with ADRs observed for bevacizumab by SOCs.

SOC	Number of Reports
Blood and lymphatic system disorders	8184
Cardiac disorders	2901
Congenital, familial and genetic disorders	155
Ear and labyrinth disorders	212
Endocrine disorders	609
Eye disorders	3386
Gastrointestinal disorders	13,456
General disorders and administration site conditions	13,360
Hepatobiliary disorders	2407
Immune system disorders	965
Infections and infestations	5915
Injury, poisoning and procedural complications	6376
Investigations	6554
Metabolism and nutrition disorders	2323
Musculoskeletal and connective tissue disorders	2669
Neoplasms benign, malignant and unspecified (incl cysts and polyps)	5336
Nervous system disorders	7851
Pregnancy, puerperium and perinatal conditions	21
Product issues	294
Psychiatric disorders	887
Renal and urinary disorders	4217
Reproductive system and breast disorders	584
Respiratory, thoracic and mediastinal disorders	7026
Skin and subcutaneous tissue disorders	3680
Social circumstances	66
Surgical and medical procedures	366
Vascular disorders	7285

## Data Availability

Data are contained within the article.
